# Alternative Controlling Agent of *Theobroma grandiflorum* Pests: Nanoscale Surface and Fractal Analysis of Gelatin/PCL Loaded Particles Containing *Lippia origanoides* Essential Oil

**DOI:** 10.3390/nano12152712

**Published:** 2022-08-07

**Authors:** Ana Luisa Farias Rocha, Ronald Zico de Aguiar Nunes, Robert Saraiva Matos, Henrique Duarte da Fonseca Filho, Jaqueline de Araújo Bezerra, Alessandra Ramos Lima, Francisco Eduardo Gontijo Guimarães, Ana Maria Santa Rosa Pamplona, Cláudia Majolo, Maria Geralda de Souza, Pedro Henrique Campelo, Ştefan Ţălu, Vanderlei Salvador Bagnato, Natalia Mayumi Inada, Edgar Aparecido Sanches

**Affiliations:** 1 Laboratory of Nanostructured Polymers (NANOPOL), Federal University of Amazonas (UFAM), Manaus 69067-005, AM, Brazil; 2 Graduate Program in Materials Science and Engineering (PPGCEM), Federal University of Amazonas (UFAM), Manaus 69067-005, AM, Brazil; 3 Amazonian Materials Group, Federal University of Amapá (UNIFAP), Macapá 68903-419, AP, Brazil; 4 Laboratory of Nanomaterials Synthesis and Nanoscopy (LSNN), Federal University of Amazonas (UFAM), Manaus 69067-005, AM, Brazil; 5 Analytical Center, Federal Institute of Education, Science and Technology of Amazonas (IFAM), Manaus 69020-120, AM, Brazil; 6 São Carlos Institute of Physics (IFSC), University of São Paulo (USP), São Carlos 13563-120, SP, Brazil; 7 EMBRAPA Western Amazon, Manaus AM-010 Km 29, Manaus 69010-970, AM, Brazil; 8 Department of Food Technology, Federal University of Viçosa (UFV), Viçosa 36570-900, MG, Brazil; 9 The Directorate of Research, Development and Innovation Management (DMCDI), Technical University of Cluj-Napoca, 15 Constantin Daicoviciu St., 400020 Cluj-Napoca, Cluj County, Romania; 10 Hagler Institute for Advanced Studies, Texas A&M University, College Station, TX 77843, USA

**Keywords:** *Lippia origanoides*, *Theobroma grandiflorum*, controlling agent, nanoscale surface, fractal analysis, controlled release, *Conotrachelus humeropictus*, *Moniliophtora perniciosa*

## Abstract

A new systematic structural study was performed using the Atomic Force Microscopy (AFM) reporting statistical parameters of polymeric particles based on gelatin and poly-*ε*-caprolactone (PCL) containing essential oil from *Lippia origanoides*. The developed biocides are efficient alternative controlling agents of *Conotrachelus humeropictus* and *Moniliophtora perniciosa*, the main pests of *Theobroma grandiflorum*. Our results showed that the particles morphology can be successfully controlled by advanced stereometric parameters, pointing to an appropriate concentration of encapsulated essential oil according to the particle surface characteristics. For this reason, the absolute concentration of 1000 µg·mL^−1^ (P_1000_ system) was encapsulated, resulting in the most suitable surface microtexture, allowing a faster and more efficient essential oil release. Loaded particles presented zeta potential around (–54.3 ± 2.3) mV at pH = 8, and particle size distribution ranging from 113 to 442 nm. The hydrodynamic diameter of 90% of the particle population was found to be up to (405 ± 31) nm in the P_1000_ system. The essential oil release was evaluated up to 80 h, with maximum release concentrations of 63% and 95% for P_500_ and P_1000_, respectively. The best fit for the release profiles was obtained using the Korsmeyer–Peppas mathematical model. Loaded particles resulted in 100% mortality of *C. humeropictus* up to 48 h. The antifungal tests against *M. perniciosa* resulted in a minimum inhibitory concentration of 250 µg·mL^−1^, and the P_1000_ system produced growth inhibition up to 7 days. The developed system has potential as alternative controlling agent, due to its physical stability, particle surface microtexture, as well as pronounced bioactivity of the encapsulated essential oil.

## 1. Introduction

The increasing interest in biodegradable particles has accelerated their development process for new technological applications [1,2,3,4,5], particularly in environmentally friendly polymeric particles containing encapsulated essential oils [6,7,8,9,10,11,12,13]. 

The combination of biomaterials with different physicochemical properties has allowed the development of layered particles, to protect and release secondary metabolites [14,15,16,17]. The evaluation of surface nanotexture and fractal analyses through Atomic Force Microscopy (AFM) technique has been useful to investigate the influence of texture parameters on the controlled release mechanism and concentration of encapsulated bioactive compounds [18,19].

Essential oils have long been considered as alternative natural agents for pest control [20,21,22]. *Lippia origanoides* Kunth [23] is popularly known as “Erva-de-Marajó” in northern Brazil. Carvacrol and thymol (the major constituents of its essential oil) present significant chemopreventive properties [24,25,26], antimicrobial activity against several pathogen groups [27], as well as repellency and a low toxicity [28]. The encapsulation of essential oils for controlled release formulations can improve their efficiency and reduce environmental damage [29,30].

The cupuaçu tree (*Theobroma grandiflorum* (Willd. *ex* Spreng.) K. Schum.) (Malvaceae) is one of the main fruit trees cultivated in the Brazilian Amazon. The high commercial value of the cupuaçu pulp derives from the food industry, mainly as juice, liqueur, and jelly, as well as in the manufacture of chocolate (“cupulate”) from its almonds [31,32,33]. *Conotrachelus humeropictus* Fiedler, 1940 (Coleoptera: Curculionidae), known as “Broca-do-Cupuaçu”, is the main pest of this culture in the Amazon region, especially in Rondônia and Amazonas [34]. This pest is difficult to control, as both the egg and larva are lodged in galleries inside the fruits. Infested fruits fall off before ripening or have the pulp completely destroyed [35]. Moreover, from the phytosanitary point of view, the disease caused by the fungus *Moniliophtora perniciosa* (known as “Vassoura-de-Bruxa”) [36] represents the main limiting factor to the expansion of this fruit tree. This pest significantly reduces the economic production, and phytosanitary pruning is the main economic tool to control this pest [37].

The use of nanotechnology to control pests in agriculture has resulted in nanoscale materials able to enhance the stability and activity of natural controlling agents [38,39]. Reports on the encapsulation of *L. origanoides* essential oil in biodegradable particles to control *C. humeropictus* and *M. perniciosa* have not been found in the scientific literature. For this reason, particles based on gelatin and poly-ε-caprolactone (PCL) were loaded with this essential oil, aiming at the development of a controlled release formulation.

The AFM technique allowed understanding the influence of the essential oil concentration on statistical parameters (based on nanoscale surface and fractal analyses), such as roughness, peak/height distributions, and nanotexture homogeneity. Size distribution measurements and nanoparticle surface charge were evaluated, respectively, by nanoparticle tracking analysis (NTA) and zeta potential. Laser Scanning Confocal Microscopy (LSCM) and fluorescence measurements were applied to confirm the essential oil encapsulation. Encapsulation Efficiency (EE%) was measured by UV-VIS spectroscopy, and the release kinetics of the essential oil was analyzed as a log cumulative percentage of released essential oil *versus* log time by fitting the data according to the Higuchi [40] and Korsmeyer–Peppa’s [41] mathematical models. Finally, the insecticidal and fungicidal efficiency of the developed formulation was assessed in vitro, respectively, against *C. humeropictus* and *M. perniciosa*.

## 2. Materials and Methods

### 2.1. Nanoparticle Development and Essential Oil Encapsulation

Colloidal system development was based on previous reports with marginal modification [14]. *L. origanoides* (SISGEN authorization code AD0C7DB) essential oil was encapsulated at absolute concentrations of 500 µg·mL^−1^ (P_500_) and 1000 µg·mL^−1^ (P_1000_). Unloaded particles (P_0_) were also prepared.

Encapsulation Efficiency (EE%) was evaluated on a Epoch2 Microplate Reader Biotek (Agilent, CA, USA) [42]. From the calibration curve, the unknown concentration of essential oil was obtained by measuring the absorbance values at 278 nm. Particles were separated by centrifugation (Daiki Sciences, Seoul City, Republic of Korea) (20,000 rpm) and the supernatant absorbance allowed obtaining the percentage of free essential oil. Then, EE% was calculated using the formula: (EE%) = (amount of encapsulated essential oil/total amount of essential oil used in the formulation) × 100. Experiments were carried out in triplicate.

### 2.2. Zeta Potential and Nanoparticle Tracking Analysis (NTA)

Zeta potential values (in mV) were obtained from pH = 1 to 10 at 25 °C. Measurements were performed in triplicate. Size characterization was performed on a NanoSight NS300 device (Malvern Instruments, Malvern, UK), equipped with a green laser type and a SCMOS camera. Data collection and analysis were performed using the software NTA 3.0 (Malvern Instruments, Malvern, UK). Samples were diluted in MilliQ water (1:100 *v*/*v*). A standard operating procedure was created using 749 frames for 30 s. Measurements were performed in triplicate. The evaluation of the particle size distribution (PSD) was performed using the parameters Mean, Mode, SD, D_10_, D_50_ (Median), and D_90_, which indicate, respectively, the average, most frequent particle class size, standard deviation, and the 10%, 50%, and 90% percentiles of the analyzed particles.

### 2.3. Laser Scanning Confocal Microscopy (LSCM) and Fluorescence Measurements

Images were taken on a Carl Zeiss microscope (inverted model LSM 780) (ZEISS Research Microscopy Solutions, Jena, Germany), with a Ti: Sapphire LASER, a 40× objective lens, 1.2 NA, and a 0.28 mm work distance. Systems containing unloaded (P_0_) and loaded particles (P_500_ and P_1000_) were centrifuged. A volume of the supernatant was discarded for visualization of the largest particles. Then, a few drops were placed on a microscopy glass slide. Measurements of fluorescence were carried out with a 63× objective and SPAD (single photon avalanche diode) detector, with a temporal resolution of 70 ps. A Coherent Chameleon tunable 690–1100 nm laser was used as the excitation source. Measurements were taken at 800 nm.

### 2.4. Atomic Force Microscopy (AFM)

Colloidal systems containing unloaded (P_0_) or loaded particles ((P_500_ and P_1000_); 1 µL) were dripped on a glass slide and dried using liquid nitrogen. Then, the glass slides containing the formed films were fixed on a sample holder using a double-sided adhesive tape. Measurements were performed at room temperature (296 ± 1 K) and (40 ± 1)% R.H. on a Innova equipment (Bruker, MA, USA) operated in tapping mode and equipped with a silicon tip and Al coated cantilever with a spring constant of 42 N/m (Tap190AL-G) (Budget Sensors, Sofia, Bulgaria). Scans were performed using (10 × 10) µm^2^ with (256 × 256) pixels at a scan rate of 1 Hz.

#### 2.4.1. Nanoscale Surface Analysis

Topographic images obtained were processed using the commercial MountainsMap^®^ software version 8.0 (Besançon, France) [43]. Stereometric parameters of height, Sk, and volume were obtained. In addition, quantitative parameters obtained from qualitative renderings (such as furrows and contour lines) were also obtained.

#### 2.4.2. Fractal Analysis

Fractal analysis was carried out based on the following superficial statistical parameters: fractal dimension (FD), surface entropy (H), fractal succolarity (FS), and fractal lacunarity (FL). Fractal dimension (FD) is commonly used for quantification of surface texture homogeneity, as well as for surface complexity evaluation. However, an analysis based only on the FD parameter is not sufficient to evaluate aspects of general texture [44], because the surface irregularity usually increases as a function of FD [45]. The free software Gwyddion 2.55 [46] (Brno, Czech Republic) was used to perform calculations.

Surface entropy (H) values quantify the uniformity of the height distribution by relating pixels and heights as a function of intensity. Measurements are based on the Shannon’s entropy (Equation (1)) [47]:

(1) $$H^{\left(2\right)}=-\sum_{i=1}^{N}\sum_{j=1}^{N}p_{ij}\cdot \log p_{ij}$$

where *p_ij_* represents the probability of finding accessible pixels on the evaluated pixel set. The AFM image (pixel matrix) was converted into a binary height matrix using the free software WSXM (Madri, Spain) [48]. Results were normalized using Equation (2) [49]:

(2) $$H_{matr^{}alt}=\frac{H^{\left(2\right)}-H_{\min}^{\left(2\right)}}{H_{\max}^{\left(2\right)}-H_{\min}^{\left(2\right)}}$$

where $H_{\max}^{\left(2\right)}$ and $H_{\min}^{\left(2\right)}$ represent, respectively, the uniform and non-uniform pattern surface (adopting the symbol H as the normalized value of surface entropy). A *R* language algorithm was programmed for H calculation using the free software RStudio 1.2.503 (Boston, MA, USA). 

Additional algorithms in *R* language and Fortran 77 were developed to obtain fractal succolarity (FS) and fractal lacunarity (FL). Percolation can be quantified through the FS evaluation (Equation (3)) [50], while FL measures the texture homogeneity by dimensioning gaps on the fractal object surface [51].

(3) $$FS(T(k),dir)=\frac{\sum_{k=1}^{n}P_{0}(T(k))\cdot PR(T(k),p_{c})}{\sum_{k=1}^{n}PR(T(k),p_{c})}$$

where *d_ir_* represents the liquid entrance direction; *T(k)* are boxes of equal size *T(n)*; *P*_0_*(T(k))* is the occupancy percentage; *PR* represents the occupancy pressure, and *p_c_* is the centroid position (x,y). FL was obtained from a previous report [52]. Calculations were focused on the lacunarity coefficient according to Equation (4) [53]:

(4) $$L(r)=\alpha \cdot r^{\beta }$$

where *L(r)* is the lacunarity, *α* represents an arbitrary constant, and *r* is the box size. The lacunarity coefficient *(β)* was estimated by *log(r)* vs. *log [*1 *+ L(r)]*. The free software Force 3.0 (Maribor, Slovenia) [54] was applied for compiling the FL algorithm. Displacement of one unit was applied, due to the small FL values.

### 2.5. Essential Oil Release

A colloidal system containing loaded particles (15 mL) was inserted in dialysis tubing cellulose membrane and suspended in water (85 mL) at 25 °C. The system was maintained under continuous magnetic stirring (100 rpm). A 3 mL aliquot was withdrawn from flask at regular time intervals (up to 80 h). Absorbance was measured at 278 nm on a Epoch2 Microplate Reader Biotek. The amount of released essential oil was calculated from a standard curve [55]. The cumulative release (%) of essential oil was obtained with the following equation: [Cumulative release (%) = (amount of essential oil released after time *t*/total amount of encapsulated essential oil) × 100]. Experiments were carried out in triplicate.

### 2.6. Insecticidal and Fungicidal Bioassays

#### 2.6.1. *Conotrachelus humeropictus*

*C. humeropictus* individuals were obtained from stock colonies at the EMBRAPA Amazônia Ocidental, Manaus/AM, Brazil, without any pesticide exposure. Borers were reared on a diet of sugarcane and kept at 25 °C, with 70–85% R. H., and a 10:14 h light:dark photoperiod. Glass Petri plates (150 mm in diameter × 20 mm in height) were used as chambers.

Filter paper (150 mm in diameter) was placed in the glass Petri dishes. Each concentration (1 mL) of essential oil in natura/acetone solution (125, 250, 500, 625, 750, and 1000 µg·mL^−1^) was uniformly applied on filter paper disk. The treated filter paper disks were air-dried for 1 min to remove solvent. Five adults were transferred from stock to the paper disk, allowing direct contact with the essential oil. Then, chambers were sealed to prevent essential oil evaporation. Acetone (1 mL) was used as negative control. Mortality was evaluated after 24 h of exposure. Individuals were considered dead if they did not move when prodded with a fine paintbrush. The experimental design was completely randomized, with three replicates. Mortality data were subjected to PROBIT analysis [56]. Then, the LD_50_ (lethal dosage that kills 50% of the exposed borers), LD_90_ (lethal dosage that kills 90% of the exposed borers), LCL (lower confidence limit) and UCL (upper confidence limit) were estimated [57], with a fiducial limit of 95%.

The toxicity of the encapsulated essential oil was also tested against *C. humeropictus*. Filter paper (150 mm in diameter) was placed in the glass Petri dishes. A volume of 1 mL of loaded particles (P_1000_) was uniformly applied on the filter paper disk. Five adults were transferred from stock to the paper disk, allowing direct contact with the loaded particles. Then, chambers were sealed to prevent loss of essential oil. Unloaded particles (P_0_) were used as negative control. The number of live borers was counted after 24 h of application. The experimental design was completely randomized based on three replicates.

#### 2.6.2. *Moniliophtora perniciosa*

*M. perniciosa* isolates were provided by the EMBRAPA Amazônia Ocidental, Manaus/AM-Brazil. Bioassays were performed by the disk diffusion method (DDM) adapted from previous report [58]. The culture medium was prepared with potato-dextrose-agar (PDA; 15.6 g) and sucrose (8.0 g), using 400 mL of distilled water, and kept under heating until complete solubilization. Essential oil was diluted in DMSO (1:9 *v*/*v*). Different volumes (1, 0.75, 0.5, 0.25, and 0.125 mL) were added to 100 mL of culture media and then transferred to Petri dishes (90 mm in diameter × 10 cm in height). All Petri dishes were inoculated with a mycelial disc (5 mm diameter) of *M. perniciosa*. Then, the Petri-dishes were incubated for 7 days at 25 °C and the colony diameter was measured. DMSO was used in the bioassays instead of essential oil as a negative control. Four replicate plates were used for each treatment.

The Minimum Inhibitory Concentration (MIC) was interpreted as the lowest concentration that inhibited visual growth. Only plates with positive growth and quality control for purity and colony counts were considered. The mycelial growth index was obtained as the ratio of the final average growth diameter to the number of days after inoculation. The relative mycelial growth percentage (RMG%) at each tested concentration was calculated by comparing the growth on amended media (GOA) compared with the growth on the nonamended control (GOC), as follows: RMG% = (GOA/GOC) × 100. The percentage inhibition of mycelium growth at each tested concentration (I) was also calculated as the difference between the radial growth of nonamended control (C) and the radial growth of each tested concentration (T), as follows: I (%) = (1 − T/C) × 100 [59].

The efficiency of the loaded particles (P_1000_) was tested against *M. perniciosa,* according to the same procedure as describe above. Unloaded particles (P_0_) were used as negative control. Four replicate plates were considered for each treatment.

## 3. Results and Discussion

### 3.1. AFM Analysis

The morphology of gelatin/PCL particles has been extensively studied in controlled release systems for pest control [14,15,17], scaffolds [60,61], and curatives [62]. Here we focused on the particles surface morphology (unloaded and loaded with *L. origanoides* essential oil), which previously showed significant larvicidal, acaricidal, and insecticidal potential [8,14,15].

**Figure 1:**
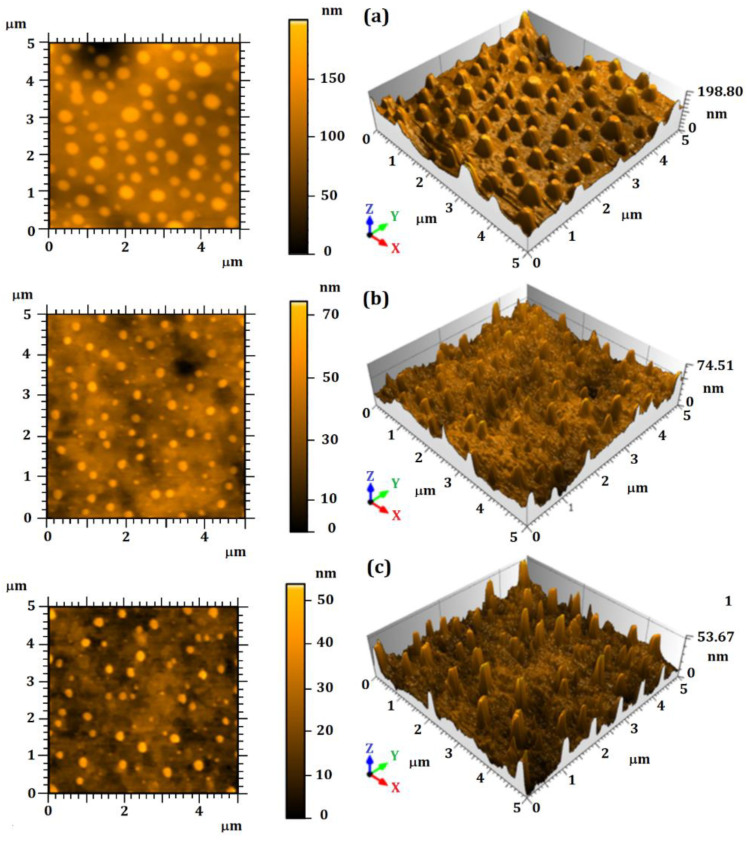
Two-dimensional and three-dimensional AFM micrographs: (**a**) unloaded particles (P_0_), (**b**) loaded particles using 500 µg·mL^−1^ of essential oil (P_500_), and (**c**) loaded particles using 1000 µg·mL^−1^ of essential oil (P_1000_).

Figure 1 shows the 3D topographic images of the unloaded particles (P_0_), as well as the particles loaded with 500 µg·mL^−1^ (P_500_) and 1000 µg·mL^−1^ (P_1000_) of essential oil. The P_0_ surface presented spherical-conical grains (Figure 1a). A thinning of the rough peaks in the loaded particles (Figure 1b,c) was observed due to the encapsulation of essential oil. Furthermore, the formation of a large spherical protuberance on the P_500_ and P_1000_ surfaces was observed, probably due to the formation of air bubbles during the drying procedure. This phenomenon was also previously observed [63]. In addition, the topography qualitative analysis revealed a different surface morphology: the increasing of the essential oil concentration promoted a smoothing on the particles surface. This behavior was confirmed by the related height surface parameters analysis (Sa and Sq), as shown in Table 1.

The results were expressed as the mean value and standard deviation, where significant difference was observed (*p*-value < 0.05). The highest roughness value was observed in P_0_ (Sq = (20.301 ± 3.030) nm). However, the Tukey test showed that both P_500_ and P_1000_ presented similar roughness values. Lower-roughness particles can present higher adhesion energy and be faster adsorbed on another surface [64]. This result indicates that the developed loaded systems represent a viable alternative to decrease particles surface roughness through the encapsulation of essential oil.

The P_0_, P_500_, and P_1000_ systems presented positive asymmetric height distributions, with Rsk values slightly greater than zero. However, the asymmetric height distribution increased in P_1000_, showing that the height distribution was affected by the increase of the essential oil concentration (although the Tukey test also revealed no significant difference between P_500_ and P_1000_). Greater asymmetry, whether positive or negative, suggests that a particle is more likely to be anchored or adsorbed onto another surface (probability because skewness is an index). This fact was observed because the particle created a preferential slope direction of its rough peaks (as observed in P_1000_). In addition, all systems also showed a non-platykurtic pattern (Leptokurtic), as the Rku values were greater than 3. Consequently, the data distribution tended to deviate from the normal Gaussian behavior [65]. As shown in Table 1, the P_1000_ system presented the highest Rsk value, differing from P_500_ (*p*-value < 0.05). These data showed that P_1000_ presented a sharper distribution, confirming its greater tendency to be easily adsorbed on another surface.

**Figure 2:**
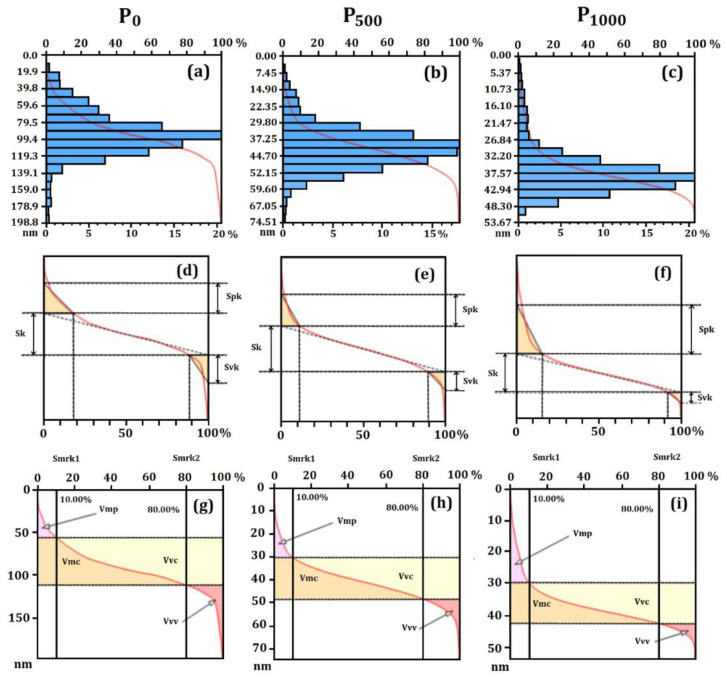
Sk values and volume parameters concerning the height distribution of the particle surface. (**a**–**c**) Particle surface of all systems (P_0_, P_500_, and P_1000_) presenting a heavy-tailed distribution (Leptokurtic) with great tapering of the height distribution; (**d**–**f**) thickness of material on the particles surface, evaluated by the height distribution according to the Sk parameter family; (**e**,**f**) displacements of the Sk curve; and (**g**–**i**) graphic behaviors considering the volume parameters of the particles surface.

Figure 2 shows the Sk values and volume parameters concerning the height distribution of the particle surface [66,67].

Figure 2a–c indicates that the particle surface of all systems (P_0_, P_500_ and P_1000_) presented a heavy-tailed distribution (Leptokurtic), with great tapering of the height distribution (mainly in P_1000_). On the other hand, the cumulative curve of Figure 2b (in red) showed better height distribution in P_500_, since approximately 90% of the relative heights were found between 0 and 0.2568 nm.

Figure 2g–i shows the graphic behaviors considering the volume parameters of the particles surface. As a result of the decrease of surface roughness, especially in P_0_ and P_500_, the volume of material forming the surface topography decreased, as observed by the peak material volume (Vmp), core material volume (Vmc), dale void volume (Vvv), and core void volume (Vvc) parameters. Statistical similarity between P_500_ and P_1000_ was also identified in all parameters. This result confirms that the topography was affected by the encapsulation of essential oil. Furthermore, the particle morphology could be controlled from the observation of advanced stereometric parameters, which could be useful for quality control of the developed material, since they accurately determined the amount of material on the particle surface in different aspects [67].

The thickness of material on the particles surface was evaluated by the height distribution according to the Sk parameter family (Figure 2d–f and Table 2). Most of the thickness and volume stereometric parameters exhibited a statistically significant difference (*p*-value < 0.05), except the valley material portion (Smr2). However, the Tukey test showed that the core thickness (Sk) values were similar to those of P_500_ and P_1000_, while the highest S_k_ value was observed in P_0_, whose behavior followed that of the surface roughness.

Similarly, the reduced peak height (Spk) and reduced valley depth (Svk) also exhibited similar behavior for P_500_ and P_1000_, showing that the thickness of the material forming the particle topography did not change from P_500_ to P_1000_. Figure 2e,f shows the displacements of the Sk curve. In addition, they also suggested that the peak material portion (Smr1) was similar in P_500_ and P_1000_. These results indicated that the surface microtexture of the particles loaded with essential oil was similar, but still without considering the complexity of the spatial patterns.

### 3.2. Surface Microtexture

**Figure 3:**
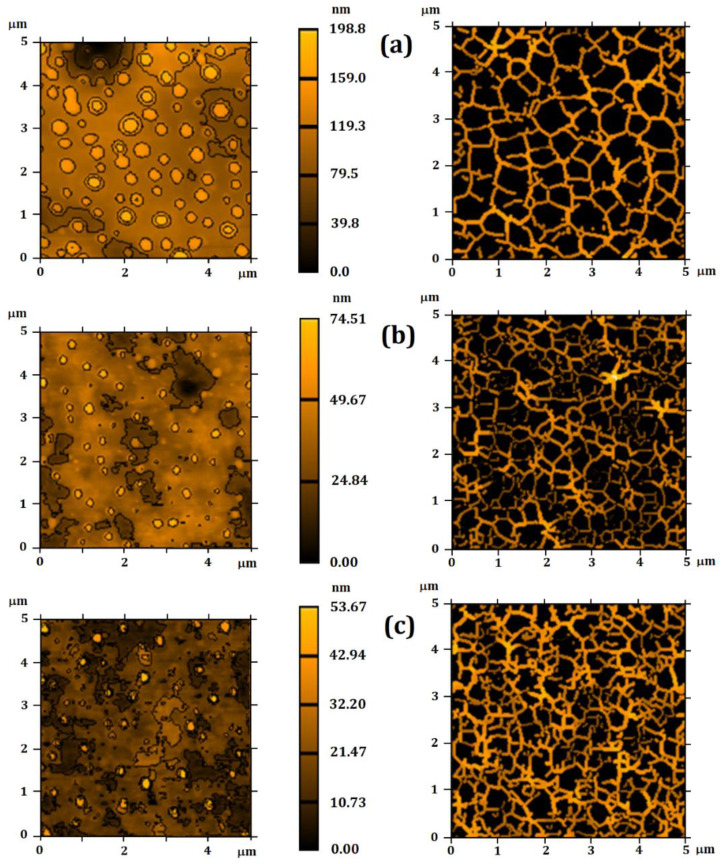
Renderings of the particle surface microtexture. Particles presented similar shapes in (**a**) P_0_, while (**b**) P_500_ and (**c**) P_1000_ acquired smaller and more randomized sizes.

Renderings of the particles surface microtexture are shown in Figure 3. Images based on furrows and contour lines were obtained for each system. This type of image has been widely used to explain the surface behavior in fluid flooding [68,69], as qualitative renderings that simulate the entrance of fluids and particle arrangement on a nanoparticle surface [52]. A significant reduction in particle size, due to the encapsulation of essential oil, was observed, which was also associated with the decreasing roughness.

Particles presented similar shapes in P_0_ (Figure 3a), while P_500_ and P_1000_ (Figure 3c) acquired smaller and more randomized sizes. These results showed that the essential oil encapsulation reduced the particle size, which could result in a better and faster adsorption of the particles on their external environment.

The regions of the images presenting more intense colors are associated with rough peaks, and the darker regions are related to valleys. All parameters associated to furrows presented statistically significant differences (*p*-value < 0.05). However, the Tukey test showed that P_500_ and P_1000_ presented a similar behavior, exhibiting shallower furrows. These data showed the decrease of the surface roughness.

A similar configuration was also observed for the mean depth of furrows (Table 3). However, P_0_ exhibited a lower mean density than those of P_500_ and P_1000_, showing that the thinning of the rough peaks promoted a greater density of furrows, and suggesting that fluids may have a greater mobility across the particle. In addition, the contour lines of the renderings revealed that the thickness of the central part of the image affected the lines distribution, probably due to the irregular relief of those surfaces.

According to these results, P_500_ e P_1000_ can be more easily penetrated by fluids, explaining the greater empty material volume in the central part of that surface.

**Figure 4:**
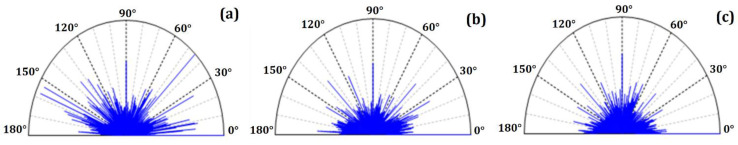
Surface texture directions for (**a**) P_0_, (**b**) P_500_, and (**c**) P_1000_. All systems presented a similar microtexture, as the direct texture parameters did not show any statistically significant differences (*p*-value > 0.05).

All systems presented similar microtexture (Figure 4), because the direct texture parameters (Table 4) did not show a statistically significant difference (*p*-value > 0.05). Although the particles presented different morphologies, the texture distribution of the topographic patterns was similar. However, such analysis is still too qualitative to propose a specific system presenting the most uniform texture, because it does not take into account the evaluation of the spatial complexity of the surface roughness distribution, which was explored by the fractal parameters.

### 3.3. Advanced Fractal Parameters

The fractal behavior of the particle surface was also evaluated, to obtain more quantitative information on the homogeneity of the microtexture. Microtexture evaluation using fractals and other related parameters has been extensively reported [70,71]. Since a fractal behavior has been attributed to objects in nature [44], several reports have focused on fractal theory to evaluate texture behavior in micro and nanoscales [72,73,74].

Table 5 presents the parameters fractal dimension (FD), surface entropy (H), fractal succolarity (FS), and lacunarity coefficient (β). FD is the first quantitative parameter associated with texture homogeneity. The fractal dimension presented similar values (*p*-value > 0.05), suggesting similar spatial complexity in all systems. For this reason, the surface microtexture was similar in P_500_ and P_1000_, although showing different morphology. However, β was smaller in P_1000_, suggesting more homogeneous surface microtexture. It is likely that the decrease of the surface roughness promoted the organization of surface gaps, resulting in a more homogeneous surface pattern for the system containing higher concentrations of essential oil. This homogeneity of the surface texture can allow a uniform mobility of fluids, improving its adsorption and release of essential oil.

On the other hand, the surface entropy analysis revealed that, although P_500_ presented more uniform height distribution (H~0.95), all particles exhibited H ≥ 0.9 (*p*-value > 0.05). According to a previous report [49], surfaces with a H higher than 0.9 are significantly uniform, indicating that both P_500_ and P_1000_ can present similar adhesion and adsorption properties, although only P_1000_ presented a more homogeneous microtexture.

Although the FS values presented a significant difference (*p-*value < 0.05), the Tukey test revealed that P_500_ and P_1000_ were similar and could be equally penetrated by fluids. These values were close to 0.5, which is considered the ideal surface percolation value [50]. Adsorption and adhesion processes on other surfaces can also be influenced, as the entrances (allowing the interaction of ligand receptor sites between surfaces) are highly dependent on the surface texture [64]. Thus, it is important to obtain an FS value lower or close to 5, so that the encapsulated systems can release the essential oil in a controlled manner (as found in P_500_ and P_1000_). These results revealed that the fractal parameters corroborated the results found in the stereometric parameters. However, the fractal lacunarity showed that P_1000_ presented the most suitable surface microtexture for adhesion to another surface, suggesting that this system could release the essential oil faster and more efficiently. For this reason, only the system P_1000_ was considered in further analyses.

### 3.4. Zeta Potential and Nanoparticle Tracking Analysis (NTA)

Zeta potential as a function of pH and NTA analysis was evaluated for the systems P_0_ and P_1000_. Zeta potential represents an important parameter for the evaluation of surface charge; besides, it is directly related to the colloidal system, influencing the particle size distribution and stability [15]. Furthermore, higher values (in module) of zeta potential are related to significant repulsion and reduction of aggregation/agglomeration [75].

A higher surface charge was found from pH ≥ 7 in the P_0_ system (data not shown), allowing formulation stability. The surface charge ranged from (–5.0 ± 0.3) mV in pH = 7 to (–12.0 ± 0.8) mV in pH = 10. The isoelectric point was verified as close to pH = 4 and was related mainly to the type B gelatin carrier. It is known that two types of gelatin (A or B) can be produced, depending on the collagen pre-treatment [76].

The particles loaded with essential oil (P_1000_) presented zeta potential values around (–54.3 ± 2.3) mV in pH = 8. The higher surface charge (in module) of the loaded particles can be attributed to the presence of the essential oil. The increased charges may be related to the compounds used to produce the particles and also to rearrangements among the essential oil constituents. The presence of these constituents probably resulted in an improved stabilization, due to new intermolecular interactions [15]: the surface electrostatic charge of particles can be influenced by several factors, including surface functional groups and solution ions [77]. On the other hand, electrostatic stability occurs due to the repulsion between particles, resulting from their high surface charge, never reaching the isoelectric point [78]. Thus, values equal to or greater than 30 mV (in modulus) are important for formulation stability [79]. For this reason, the surface charge of the P_1000_ system was found in a range that guarantees its stability as a colloidal system.

**Figure 5:**
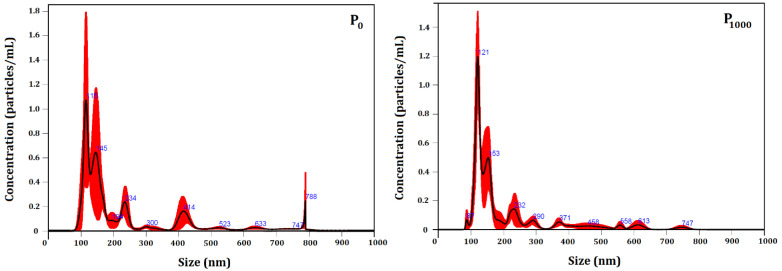
NTA particle size distribution analysis of P_0_ and P_1000_ systems. Representative histograms of the average size distribution (black line) from three measurements of a single sample. Red areas specify the standard deviation (SD) between measurements, and blue numbers indicate the maxima of individual peaks.

Unloaded (P_0_) and loaded particles (P_1000_) were characterized for number and size distribution by NTA (Figure 5). Table 6 shows the average particle size of P_0_ and P_1000_. The developed colloidal systems were compared, in terms of both size and concentration (particles/mL) as a function of encapsulated essential oil. No significant change in number of particles or in their size was observed, as registered by all the size descriptors.

The developed systems presented a polydisperse particle size distribution, ranging from 113 nm to 442 nm. Moreover, 90% of the particle population in the P_0_ and P_1000_ systems presented a size up to (442 ± 12) nm and (405 ± 31) nm, respectively.

The mode parameter shows the particle size (or size range) most commonly found in the population distribution, and it is helpful to describe the midpoint for nonsymmetric distributions [80]. The value that best represents the encapsulated particle size was (128 ± 8) nm.

Our results showed that the particle size distribution profile was not significantly influenced after the encapsulation of the essential oil. However, the presence of the essential oil in the P_1000_ system positively influenced its stability through the increase of the particle surface charge.

### 3.5. Laser Scanning Confocal Microscopy (LSCM) and Fluorescence Measurements

**Figure 6:**
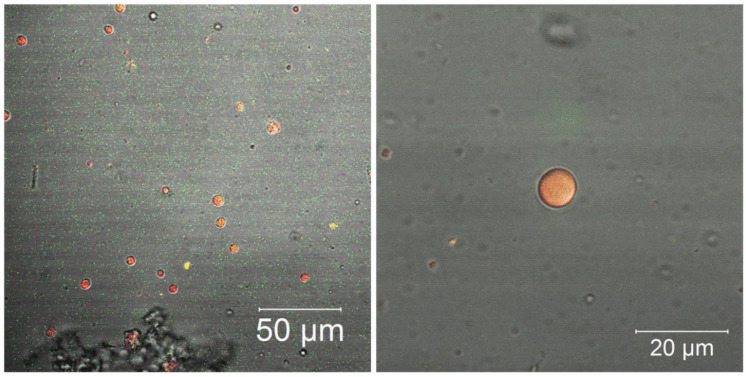
Confocal microscopy images of the particles from loaded system (P_1000_).

Figure 6 shows the particles images of the loaded particles, P_1000_. Larger particles (µm) were selected. According to the NTA measurements, 10% of the loaded particles were larger than (405 ± 31). The essential oil was homogeneously located within the loaded particles/capsules. Moreover, an absence of essential oil was observed in the unloaded system (data not shown), as expected.

Since the fluorescent properties of various molecules are highly dependent on the environment, this is a potentially useful method for determining material complexation [81]. 

**Figure 7:**
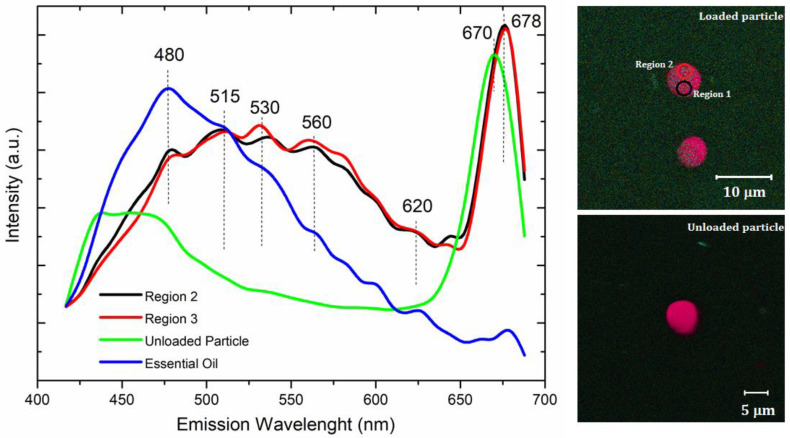
Fluorescence measurements of the loaded (regions 1 and 2) and unloaded particles.

Fluorescence measurements were performed on the unloaded and loaded particles. Emission spectra are presented in Figure 7 and show that the fluorescence intensity was mainly dependent on the essential oil. The luminance phenomenon of essential oil is caused by the *π*-electron conjugated system present in its constituents.

The loaded particles presented a sensitive fluorescence response, under the same wavelength as the free essential oil. The emission spectrum of the P_1000_ system (regions 1 and 2) presented similar peaks, mainly at 480 nm, 515 nm, 530 nm, 560 nm, 620 nm, 670 nm, and 678 nm, confirming the essential oil encapsulation. In these cases, the fluorescence of the loaded particles was observed at a definite excitation length, owing to the fluorescent of secondary metabolites encapsulated within the polymeric particles. However, the fluorescence intensity of the loaded particles increased from 515 nm to 650 nm. In this system, well-defined and more intense emission peaks were assigned to the carriers (such as gelatin and PCL) and observed mainly at 678 nm. A blue shift of this peak was observed from 678 nm to 670 nm, due to the presence of essential oil. In conclusion, the results suggested weak interactions of an electrostatic nature that connected essential oil molecules with polymeric carriers. These interactions did not cause chemical changes in the essential oil. The emission peaks of the essential oil were not observed in the P_0_ system, as expected.

Fluorescence measurements have been widely applied to evaluate chemical interactions in material complexation [82]. Similar results were observed elsewhere [83]. The composite of *bis*-eugenol/mesoporous silica presented a sensitive fluorescence response similar to that of free *bis*-eugenol obtained from clove oil. The authors suggested a weak hydrogen bond connecting the *bis*-eugenol molecules with the Si–OH groups of the silica porous wall. On the other hand, a significant enhancement of the fluorescence intensity of *Salvia sclarea* L. essential oil (SEO), due to its complexation with *β*-cyclodextrin (*β*-CD), was also investigated [82].

### 3.6. Controlled Release

The release kinetics were investigated, to understand the mechanisms of release of essential oil from the gelation/PCL particles as a function of the encapsulated concentration. Encapsulation efficiency (EE%) was found to be higher than 99% in both the P_500_ and P_1000_ systems.

**Figure 8:**
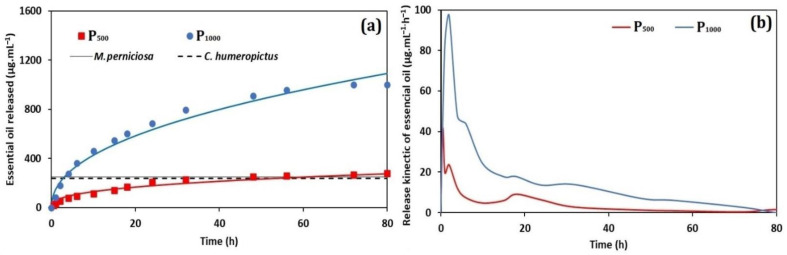
Controlled release curves of the P_500_ and P_1000_ systems: (**a**) concentration of released essential oil (µg·mL^−1^), and (**b**) kinetic essential oil release (µg·mL^−1^ h^−1^).

Figure 8a shows the profile of release of essential oil. A significant difference was observed in the released concentration of essential oil in the P_500_ and P_1000_ systems. The essential oil release was evaluated up to 80 h, with maximum release concentrations of 63% and 95% for P_500_ and P_1000_, respectively. These results agree with the fractal lacunarity values from AFM, which suggested that the P_1000_ system presented the most suitable surface microtexture for a more efficient release of essential oil. As also observed, the decrease of the surface roughness of P_1000_ resulted in a more homogeneous surface pattern. Thus, this observed homogeneity favors uniform mobility of fluids on the surface particle, as well as the solubilization of the gelatin carrier, improving its adsorption and the release of the bioactive compound. This is a possible reason for the lower concentration of essential oil released from the P_500_ system. A similar behavior of the encapsulated systems was also observed previously for gelatin/PCL particles containing essential oil from *Piper aduncum* and *Piper hispidinervum* [14].

After 24 h, the P_1000_ system released (51.5 ± 0.3)% of the total amount of encapsulated essential oil and, after 48 h, the released concentration reached (90.2 ± 0.4)%.

Figure 8b shows the derived curves from controlled release. All curves show a large release peak, representing a rapid release of essential oil in the first minutes of evaluation, resulting in the flow of essential oil into the solution. A rapid initial release followed by more sustained release was previously reported considering the essential oil of oregano in chitosan nanoparticles [84]: approximately 82% of the encapsulated essential oil was released up to 3 h. A rapid release of essential oil favors its high concentration in the medium, maintaining its effectiveness for a longer period [85].

The concentration of released essential oil observed in Figure 8a suggests that only the P_1000_ system may show effectiveness in controlling *C*. *humeropictus* and *M. perniciosa*, because their lethal dosages were reached (as shown in the next section). The profile of release of essential oil from the loaded particles was analyzed by applying the Higuchi [40] and Korsmeyer-Peppas [41] mathematical models. Linear regression was used to calculate the values of the release constants (*k*) and the correlation coefficients (R^2^). The results are summarized in Table 7.

The mathematical models presented good adjusted to the experimental curves, resulting in a R² from 0.95 to 0.99. The best fit to the release profiles of both P_500_ and P_1000_ was obtained using the Korsmeyer–Peppas mathematical model. Release profile curves were analyzed using a simple empirical model, [*f = kt^n^*] [86,87,88]. The kinetic constant *k* is a characteristic of a particular system considering structural and geometrical aspects; *n* is the release exponent representing four different mechanisms (Fickian diffusion, anomalous transport, Case-II transport, and Super Case-II transport) [89], considering spherical particles, and *t* is the release time.

The release mechanism by Fickian diffusion is the mechanism in which the active diffusion through the particle is exclusively determined by Fickian diffusion. In the case of anomalous transport, the active release is due both to Fickian diffusion and swelling/relaxation of the carrier. Case-II transport is controlled by the swelling and relaxation of carriers and it is independent of time. In Super Case-II transport, the release is ruled by the macromolecular relaxation of the polymeric chains [86].

In general, the *n* value determines the dominant release mechanism. Considering spherical particles, *n* ≤ 0.43 represents a Fickian diffusion (Case I); 0.43 ≤ *n* ≤ 0.85 represents an anomalous transport. When *n* = 0.85, the release is governed by Case-II transport, and *n* > 0.85 is related to Super Case-II transport [40].

The release assays showed that for the same period (80 h), there was a greater release of essential oil from the P_1000_ system. However, the release constant (*k*) values obtained for both P_500_ and P_1000_ systems (based on the Korsmeyer–Peppas mathematical model) showed that the release rate of the P_1000_ system (14.4 h^−1^) was slower. Furthermore, the concentration of the encapsulated essential oil influenced the release mechanism. Particles containing a higher concentration of encapsulated essential oil (P_1000_) were released according to the non-Fickian transport (*n* = 47). On the other hand, the P_500_ system presented a Fickian diffusion (Case-I) (*n* = 0.36) [90,91].

### 3.7. Insecticidal and Fungicidal Bioassays

The bioactivity of the *L. origanoides* essential oil against various pests can occur in different ways, causing mortality, deformation at different stages of development, as well as repellency [92]. Secondary metabolites have shown insect toxicity in the vapor phase, being reported as more toxic to microorganisms than in the contact form [14].

Our results indicated that the essential oil in natura presented an insecticidal activity against *C. humeropictus*. The LD_50_ was found to be around (240 ± 25) μg·mL^−1^ after 24 h of exposure, with a lower confidence limit (LCL) and upper confidence limit (UCL), respectively, of 131 μg·mL^−1^ and 350 μg·mL^−1^. The fiducial limit was considered as 95%.

The P_1000_ system was submitted to bioassays against *C. humeropictus*. Particles containing *L. origanoides* showed 100% mortality up to 48 h. About 80% of the borers were killed within 24 h. These results agree with the released concentration of essential oil from the controlled release curves and show that P_1000_ system was efficient against this tested pest, resulting in their control for up to 24 h. Similar works were found in the scientific literature considering other borer species [93,94].

The repellent activity of *Lippia origanoides*, *L. alba*, *Tagetes lucida*, *Rosmarinus officinalis*, *Cananga odorata*, *Eucalyptus citriodora*, and *Cymbopogon citratus* essential oils from Columbia were previously tested against the borer *Sitophilus zeamais* [95]. The authors observed that *L. origanoides* was found to be the most effective, causing (92 ± 3)% repellency at a 0.503 μL.cm^−2^ dose. The insecticidal activity of essential oils from *Thymus vulgaris* (thyme) and *Cymbopogon citratus* (lemongrass) against the devastating pest *Tuta absoluta* was also reported [96]. The resultant biological parameters for lemongrass and thyme oils were LD_50_ of 1479 µL·mL^−1^ and 3046 µL·mL^−1^ for lemongrass and thyme oils, respectively, considering their fumigant toxicity.

The antifungal activity of *L. origanoides* has been extensively reported [97]. Considering the concentrations of essential oil added to the culture medium (0.125, 0.25, 0.5, 0.75, and 1 mg·mL^−1^), the mycelial growth of *M. perniciosa* was observed only at 0.125 µg·mL^−1^. For this reason, the tested concentration of 250 µg·mL^−1^ was considered as the MIC value.

The bioassays presented statistically significant differences (*p*-value < 0.05) between the essential oil and control. The treatments resulted in a percentage inhibition of mycelium growth of (57 ± 8)%, as shown in Table 8.

The efficiency of the P_1000_ system was evaluated against *M. perniciosa*. After 7 days of incubation, no mycelial growth percentage was observed. Carvacrol has been reported as the major constituent of the essential oil from *L. origanoides* [23] and has shown efficiency in controlling bacteria [98,99], fungi [98,100], and insects [23,101]. The inhibition of *Phytophthora infestans*, a phytopathogen of potato, was reported with MIC = 150 µg mL^−1^, confirming the efficiency of this essential oil in controlling pathogenic fungi [102].

## 4. Conclusions

The present study successfully developed gelatin/PCL-based particles as useful carriers of the essential oil from *L. origanoides*. The proposed colloidal system can release lethal dosage concentrations to control *C. humeropictus* and *M. perniciosa* for up to 24 h, which are the main pests of *Theobroma grandiflorum*. The AFM data also showed that the encapsulation of essential oil affected the particle’s surface morphology. The surface roughness decreased as a function of the concentration of encapsulated essential oil. The homogeneity of the surface texture observed in the P_1000_ system allowed a uniform mobility of fluids on the surface, improving its adsorption and release of essential oil. These results were observed in the controlled release assays. The nanoscale surface and fractal analysis based on AFM technique represent an useful tool for quality standards in manufacturing particles containing encapsulated essential oil. For this reason, our results suggested that the developed particles containing encapsulated essential oil could be applied as a sustainable alternative controlling agent for the tested pests, combined with their biodegradability and adequate controlled release, with promising future applications.

## Figures and Tables

**Table 1:** Surface parameters (Sa, Sq, Rsk, and Rku).

Parameters	Samples		
	P_0_	P_500_	P_1000_
Sa (nm)	27.208 ± 3.030	8.032 ± 0.664	6.163 ± 1.352
Sq (nm)	20.301± 5.248	10.546 ± 1.244	8.941 ± 2.120
Rsk	0.164 ± 0.572	0.542 ± 0.064	1.406 ± 0.456
Rku	4.183 ± 0.363	4.168 ± 0.353	6.944 ± 1.009

**Table 2:** Sk and volume parameters of the particles surface.

Parameters	Systems		
	P0	P500	P1000
Sk (µm)	50.398 ± 10.360	23.140 ± 1.829	15.067 ± 2.938
Spk (µm)	39.308 ± 5.400	14.609 ± 0.269	19.946 ± 5.278
Svk (µm)	35.393 ± 13.872	8.009 ± 0.776	6.935 ± 3.758
Smr1(%)	17.842 ± 1.779	12.566 ± 1.217	14.687 ± 1.106
Smr2 (%) *	89.646 ± 1.928	90.028 ± 0.799	90.623 ± 0.703
Vmp (µm/µm2)	0.001 ± 0.000	0.001 ± 0.000	0.001 ± 0.000
Vmc (µm/µm2)	0.020 ± 0.003	0.009 ± 0.001	0.006 ± 0.001
Vvc (µm/µm2)	0.036 ± 0.001	0.012 ± 0.002	0.010 ± 0.003
Vvv (µm/µm2)	0.003 ± 0.001	0.001 ± 0.000	0.001 ± 0.000

* Samples without significant difference ANOVA one-way and Tukey test (*p*-value > 0.05).

**Table 3:** Furrow parameters (maximum depth, mean depth, and mean density).

Furrow Parameters	Systems		
	P_0_	P_500_	P_1000_
Maximum depth (µm)	78.973 ± 5.331	33.127 ± 1.762	29.623 ± 3.243
Mean depth (µm)	51.470 ± 3.118	17.722 ± 0.201	17.788 ± 1.506
Mean density (cm/cm^2^)	31,933.762 ± 1044.323	42,288.498 ± 433.281	42,358.011 ± 643.838

**Table 4:** Surface texture isotropy (STI) and the respective directions.

Time (s)	First Direction (°) *	Second Direction (°) *	Third Direction (°) *	STI (%) *
P_0_	134.995 ± 77.938	112.501 ± 38.974	88.624 ± 49.674	61.817 ± 19.551
P_500_	165.995 ± 9.578	135.321 ± 0.453	37.626 ± 7.138	64.913 ± 7.4248
P_1000_	67.503 ± 74.616	123.749 ± 37.310	112.511 ± 38.965	49.691 ± 17.423

* Samples without significant difference ANOVA One-Way and Tukey Test (*p*-value < 0.05).

**Table 5:** Fractal dimension (FD), surface entropy (H), fractal succolarity (FS), and lacunarity coefficient (β). Average results are expressed as mean values and standard deviations.

Time (s)	P_0_	P_500_	P_1000_
FD *	2.30 ± 0.03	2.266 ± 0.006	2.29 ± 0.04
H *	0.93 ± 0.04	0.95 ± 0.03	0.90 ± 0.02
FS	0.61 ± 0.04	0.52 ± 0.01	0.59 ± 0.03
׀β׀	5.74 × 10^−4^ ± 2.79 × 10^−5^	2.93 × 10^−4^ ± 6.43 × 10^−5^	1.18 × 10^−4^ ± 1.53 × 10^−5^

* Samples without significant difference ANOVA One-Way and Tukey Test (*p*-value < 0.05).

**Table 6:** Average particle size measured by NTA considering the P_0_ and P_1000_ systems.

Parameters	P_0_	P_1000_
Mean (nm)	215 ± 14	202 ± 7
Mode (nm)	122 ± 12	128 ± 8
SD (nm)	161 ± 1	134 ± 15
D_10_ (nm)	113 ± 10	113 ± 3
D_50_ (nm)	135 ± 11	141 ± 8
D_90_ (nm)	442 ± 12	405 ± 31
Concentration (particles/mL)	(6.0 ± 0.9) × 10^10^	(5.0 ± 0.6) × 10^10^

Parameters D_10_, D_50_, and D_90_ indicated that 10%, 50%, or 90% of the particle’s population, respectively, presented a diameter of less than or equal to the specified value.

**Table 7:** Coefficients obtained from the controlled release according to the Higuchi and Korsmeyer–Peppas mathematical models.

Model	Coefficient	P_500_	P_1000_
Higuchi	K	31.1	12.46
	R²	0.95	0.95
Korsmeyer–Peppas	K	57.0	14.4
	n	0.36	0.47
	R²	0.99	0.99

**Table 8:** Growth and inhibition parameters of *M. perniciosa,* considering the *L. origanoides* essential oil and the tested control.

	Diameter (mm)	RGM (%)	I (%)	MGI (mm/day)
L. origanoides	32 ± 6	43 ± 8	57 ± 8	4.6 ± 0.8
Control	74.8 ± 0.5	100.00	0.00	10.7 ± 0.1

RGM: relative mycelial growth percentage; I: percentage inhibition of mycelium growth; MGI: mycelial growth index. Negative control: DMSO.

## Data Availability

Not applicable.
